# Video‐assisted thoracoscopic surgery versus open thoracotomy for resection of lung metastasis—A meta‐analysis of reconstructed time‐to‐event data

**DOI:** 10.1111/1759-7714.15473

**Published:** 2024-10-17

**Authors:** Felipe S. Passos, Pedro B. Bregion, Luca Fazzini, Hristo Kirov, Tim Sandhaus, Patrick von Samson, Torsten Doenst, Tulio Caldonazo

**Affiliations:** ^1^ Department of Thoracic Surgery MaterDei Hospital Salvador Brazil; ^2^ Department of Cardiothoracic Surgery State University of Campinas São Paulo Brazil; ^3^ Department of Medical Sciences and Public Health University of Cagliari Cagliari Italy; ^4^ Department of Cardiothoracic Surgery Jena University Hospital Jena Germany; ^5^ Department of Cardiothoracic Surgery Weil Cornell Medicine New York New York USA

**Keywords:** lung metastasectomy, lung metastases, open thoracotomy, video‐assisted thoracoscopic surgery

## Abstract

This study aimed to conduct a systematic review and meta‐analysis comparing video‐assisted thoracoscopic surgery (VATS) and open thoracotomy (OT) in the context of pulmonary metastasectomy. Three databases were assessed. The primary outcome was overall survival. The secondary outcomes were recurrence‐free survival, ipsilateral recurrence, and hospital length of stay (LOS). Hazard ratios (HRs), odds ratios (ORs), and mean difference (MD) with 95% confidence intervals (CIs) were calculated. Reconstruction of time‐to‐event data and sensitivity analyses were performed for the primary endpoint. After screening, 11 studies were included encompassing 2159 patients undergoing lung metastasectomy (VATS: 827; OT: 1332). Compared to OT, patients who underwent VATS had higher overall survival rates (HR 0.75; 95% CI 0.67–0.85; *p* < 0.01), no significant difference in recurrence‐free survival (HR 1.07; 95% CI 0.88–1.29; *p* = 0.48), shorter hospital LOS (MD −1.99 days; 95% CI −2.59 to −1.39; *p* < 0.01), and no significant difference in ipsilateral recurrence rates (OR 0.86; 95% CI 0.52–1.42; *p* = 0.56). For patients undergoing pulmonary metastasectomy, VATS strategy is associated with higher survival rates and reduced hospital LOS when compared with OT. Moreover, metastasis recurrence does not seem to be associated with long‐term mortality in this population.

## Graphical abstract

Video‐assisted thoracoscopic surgery (VATS) is associated with improved overall survival and shorter hospital stays compared to open thoracotomy (OT) in patients undergoing lung metastasectomy, without significant differences in recurrence rates. VATS offers a less invasive alternative with comparable long‐term outcomes.

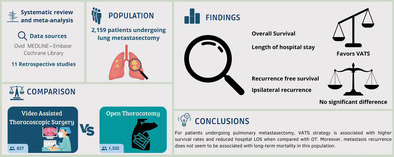

## INTRODUCTION

Lung metastases occur frequently in patients with cancer, with an increasing frequency attributed to cancer treatment advancements that have improved survival rates.^1^, ^2^, ^3^, ^4^ Resection of pulmonary metastases enhances the survival outcomes in selected cases.^1^, ^2^, ^4^, ^5^, ^6^, ^7^, ^8^, ^9^ Contemporary surgical practice has a notable trend towards minimally invasive procedures.^4^, ^10^, ^11^ Specifically, video‐assisted thoracoscopic surgery (VATS) offers a less invasive alternative with several patient benefits, including improved pain management, shorter hospitalization durations, and enhanced tolerance to postoperative chemotherapy compared to open thoracotomy (OT).^2^, ^5^, ^12^, ^13^, ^14^

Nonetheless, the success of VATS hinges on precise preoperative imaging for nodule localization, as the impossibility of palpating the entire lung may result in undetected nodules and a potential for increased recurrence rates.^4^, ^6^, ^7^, ^10^, ^11^, ^12^, ^13^, ^15^, ^16^ While OT may allow the detection of a greater number of lung nodules compared to VATS, existing evidence does not strongly support superior survival outcomes or overall benefits with this approach in pulmonary metastasectomy patients.^5^, ^11^, ^13^, ^16^ The optimal surgical approach for complete metastasectomy remains controversial, particularly when comparing VATS with OT.^3^, ^5^, ^11^, ^13^, ^16^

Therefore, this study aims to conduct a comparative analysis of the major clinical outcomes associated with VATS versus OT.

## METHODS

The study selection followed the Preferred Reporting Items for Systematic Reviews and Meta‐Analyses (PRISMA) guidelines.^17^ The review was registered in the International Prospective Register of Systematic Reviews (PROSPERO, CRD42024568526).

### Search strategy

A comprehensive literature search was performed on Ovid MEDLINE, EMBASE, and Cochrane Library to identify contemporary studies comparing outcomes between VATS and OT in patients with pulmonary metastasis published up to July 2024. We also searched for additional studies using the references of previously included studies. The complete search strategy is available in Supplementary Table 1.

### Study selection

Two independent reviewers (FP and PB) screened the records, after de‐duplication. Any discrepancies and disagreements were resolved by a third author (TC). Titles and abstracts were reviewed against pre‐defined inclusion and exclusion criteria.

### Eligibility criteria and quality assessment

Inclusion criteria for studies involved in this analysis were as follows: (1) randomized controlled trials (RCTs) or observational studies, (2) comparing VATS versus OT, (3) enrolling adult patients undergoing lung metastasectomy, and (4) reporting at least one outcome of interest. Exclusion criteria included studies involving animal models, conference abstracts, case reports, and non‐comparative study designs. The quality of included studies was assessed using the Newcastle–Ottawa Scale (NOS) for observational studies (Supplementary Table 2).^18^ Publication bias was assessed for the primary outcome.

### Data extraction

Two reviewers (FB and PB) independently performed data extraction. Accuracy was verified by a third author (TC). The extracted variables included study characteristics (publication year, study design, country, sample size, and reported outcomes) as well as patient demographics (age, sex, site of primary tumor, and sex).

### Endpoints

The primary outcome was overall survival. The secondary outcomes were recurrence‐free survival, ipsilateral recurrence, and hospital LOS.

### Statistical analysis

Odds ratio (OR) and mean difference (MD) with 95% confidence intervals (CI) were calculated for binary outcomes and continuous outcomes, respectively. Time‐to‐event data strategy was utilized for overall survival and recurrence‐free survival outcomes. Heterogeneity was assessed with Cochran *Q* test and *I* ^*2*^ statistic; *p* < 0.10 and *I* ^2^ >50% were considered significant for heterogeneity.^19^ A *p*‐value <0.05 was considered for significant difference between the two groups. DerSimonian and Laird random effects models were used for all endpoints.^20^ The Cochrane Handbook for Systematic Reviews of Interventions was used for data handling and conversion.^21^

### Individual patient survival data meta‐analysis

We used the methods described by Wei et al.^22^ to reconstruct individual patient data (IPD) from the Kaplan–Meier curves of all eligible studies for the long‐term outcomes. Raster and Vector images of the Kaplan–Meier survival curves were pre‐processed and digitized, so that the values reflecting to specific timepoints with their corresponding survival/mortality information could be extracted. Where additional information (e.g., number‐at‐risk tables or total number of events) were available, they were used to further calibrate the accuracy of the time‐to‐events. To confirm the quality of the timing of failure events captured, we thoroughly checked the consistency with the reported survival or mortality data provided in the original publications.

### Meta‐analysis of reconstructed data—one‐stage survival meta‐analysis

The Kaplan–Meier method^23^ was used to calculate the overall survival and the disease‐free survival rates. The Cox proportional hazards regression model was used to assess between‐group differences. For these Cox models, the proportional hazard assumption was verified by plotting scaled Schoenfeld residuals, log–log survival plots, and predicted versus observed survival functions. We plotted survival curves using the Kaplan–Meier product limit method and calculated the hazard ratios (HRs) and 95% CIs of each group. A HR inferior to 1 indicated higher survival rates in the VATS arm.

### Sensitivity analyses

To address the robustness of the findings, the following sensitivity analyses were performed for the primary endpoint: two‐stage meta‐analysis addressing the individual HR, leave‐one‐out analysis, Egger's test for publication bias, and a subgroup analysis addressing studies published before and after 2010 for the primary endpoint. All statistical analyses were performed using R (version 4.4.0, R Foundation for Statistical Computing, Vienna, Austria) and STATA IC17.0 (StataCorp LLC, College Station, Texas).

## RESULTS

### Study characteristics

**FIGURE 1:**
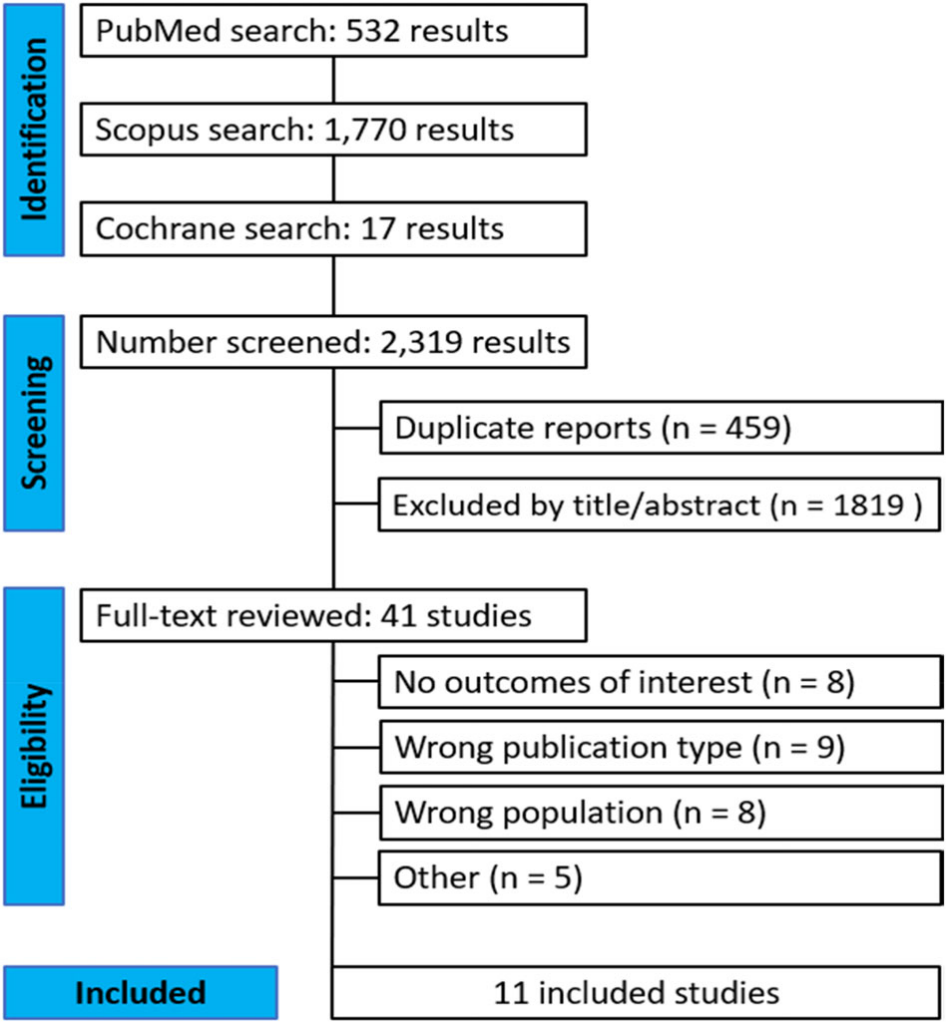
Preferred Reporting Items for Systematic Reviews and Meta‐Analyses (PRISMA) flow diagram.

Figure 1 shows the PRISMA flow diagram outlining the study selection process. A total of 2319 studies were retrieved from the systematic search, of which 11 met the criteria for inclusion in the final analysis.^1^, ^2^, ^5^, ^6^, ^7^, ^8^, ^10^, ^11^, ^12^, ^13^, ^15^ Included studies were published between 2002 and 2023. All studies used registry data and originated from Canada, China, France, Japan, Netherlands, Taiwan, UK, and United States.

### Patient characteristics

Table 1 shows the individual study information. Eleven observational studies were included in this meta‐analysis, of which 10 were retrospective and one was prospective, encompassing 2159 patients. From this group, 827 patients underwent VATS and 1332 to OT. The number of patients in each study ranged from 35 to 1047. The age ranged from 40 to 69 years, with the percentage of male patients varying from 27% to 67%.

**TABLE 1:** Study and patient's baseline characteristics of included studies.

Study	Country	Sample size VATS/OT (*n*)	Male VATS/OT *n* (%)	Age VATS/OT, years, median or mean	Primary tumor	Follow‐up, months, median, or mean
Carballo (2009)	United States	36/135	18 (50)/82 (60.7)	58.5/49^a^	Not specific	26.2^a^
Chao (2012)	Taiwan	35/35	17 (48.57)/22 (62.85)	NR/NR	Colorectal cancer	50^b^
Claramunt (2012)	Canada	75/136	43 (57.3)/75 (55.1)	63/60^b^	Colorectal cancer	NR
Gossot (2009)	France	31/29	21 (68)/13 (45)	43/40^b^	Sarcoma	34^b^
Han (2013)	Japan	62/43	37 (59.7)/29 (67.4)	58/60^a^	Not specific	36^a^
Hou (2015)	China	57/57	39 (68.42)/35 (61.4)	62/61^a^	Colorectal cancer	45^a^
Liu (2023)	Taiwan	27/16	13 (48)/11 (69)	58/59^a^	Not specific	29/28^a^, ^c^
Markowiak (2021)	Germany	63/188	42 (66.7)/124 (66)	64.9/60.4^b^	Not specific	NR
Murakawa (2017)	Japan	400/647	230 (57.5)/398 (61.51)	64.7/64.4^b^	Colorectal cancer	NR
Mutsaerts (2002)	Netherlands	16/19	8 (50)/6 (31.58)	52/61^a^	Not specific	16/25^a^, ^c^
Nakas (2009)	UK	25/27	16 (64)/19 (70)	69/66^a^	Colorectal cancer	22^a^

Abbreviations: NR, not reported; OT, open thoracotomy; VATS, video‐assisted thoracoscopic surgery.

^a^ Median.

^b^ Mean.

^c^ Data are presented as VATS/OT.

### Primary outcome

**FIGURE 2:**
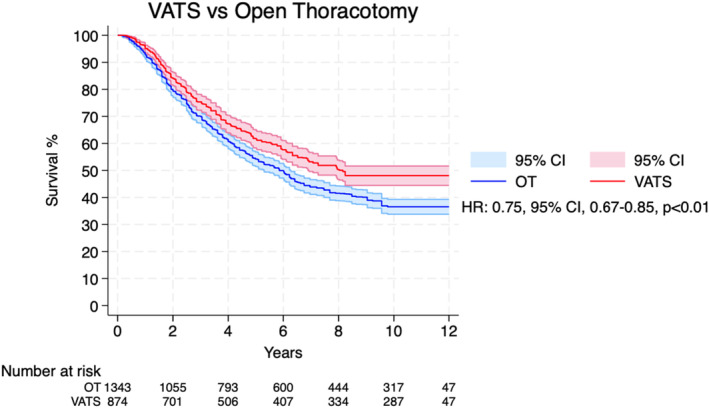
Overall survival for patients undergoing video‐assisted thoracoscopic surgery and open thoracotomy for pulmonary metastasectomy. CI, confidence interval; HR, hazard ratio; VATS, video‐assisted thoracoscopic surgery; OT, open thoracotomy.

Overall, 11 Kaplan–Meier curves were processed, digitalized, and reconstructed. Figure 2 shows the pooled Kaplan–Meier curves for the entire observation period for overall survival. The patients who underwent VATS showed higher overall survival when compared to the OT group (HR 0.75; 95% CI 0.67–0.85; *p* < 0.01; Figure 2 and Table 2).

**TABLE 2:** Summary of outcomes.

Outcome	Number of studies	Number of patients	Effect estimate (95% CI, *p*‐value)
Overall survival	11	2159	HR 0.75; 95% CI 0.67–0.85; *p* < 0.01
Recurrence‐free survival	8	831	HR 1.07; 95% CI 0.88–1.29; *p* = 0.48
Length of hospital stay	5	533	MD −1.99; 95% CI −2.59 to −1.39; *p* < 0.01
Ipsilateral recurrence	4	438	OR 0.86; 95% CI 0.52–1.42; *p* = 0.56

Abbreviations: CI, confidence interval; HR, hazard ratio; MD, mean difference; OR, odds ratio.

### Sensitivity analyses

The same tendency was detected when addressing the individual HR of the studies in the two‐stage meta‐analysis using random‐effects model (Supplementary Figure 1). The leave‐one‐out sensitivity analysis revealed that the Murakawa et al.^15^ study presents an outlier behavior compared with the other reports (Supplementary Figure 2). The funnel plot did not reveal significant asymmetry (*p* = 0.59; Supplementary Figure 3).

Finally, the subgroup analysis showed significant survival advantage for the VATS group compared to OT in the studies published after 2010 (*p*_interaction_: 0.013) and not in the studies published before 2010 (*p*_interaction_: 0.910) (Supplementary Figure 4).

### Secondary endpoints

**FIGURE 3:**
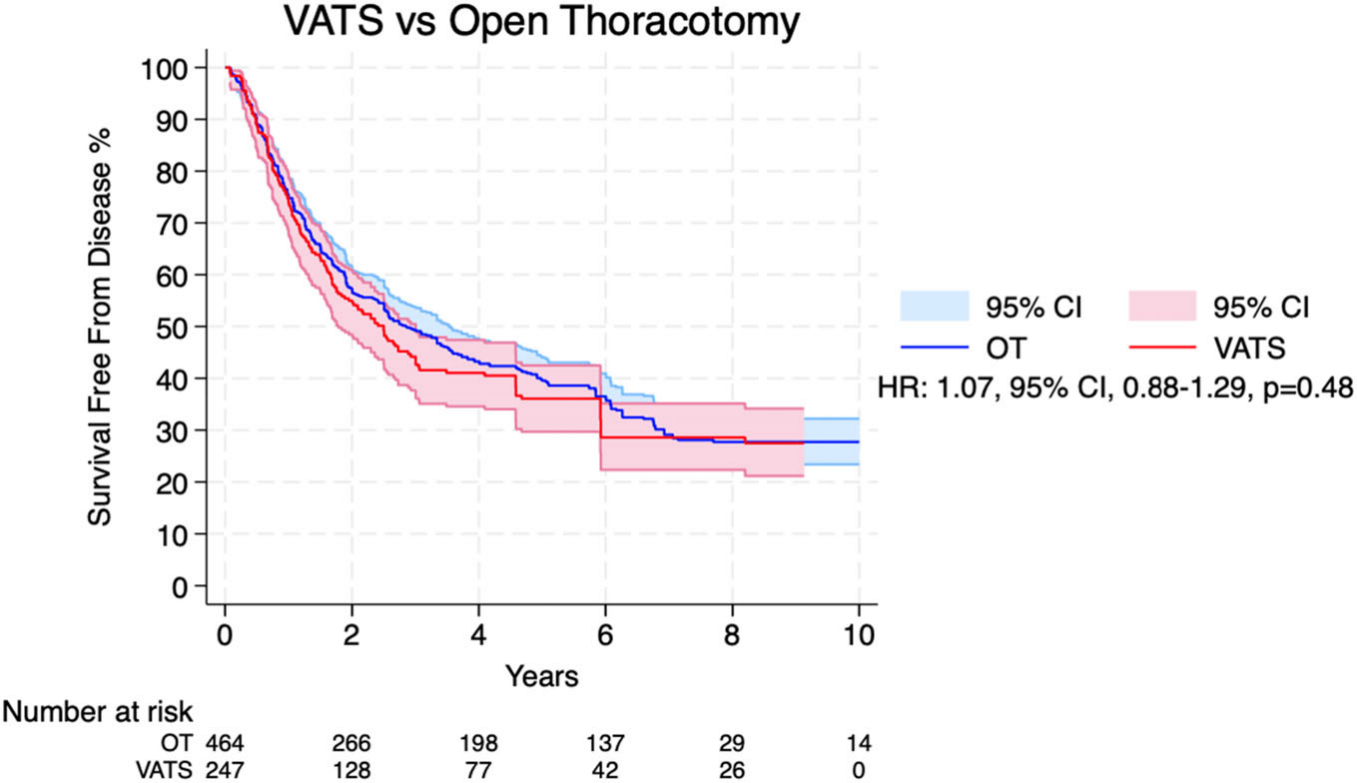
Recurrence‐free survival for patients undergoing video‐assisted thoracoscopic surgery and open thoracotomy for pulmonary metastasectomy. CI, confidence interval; HR, hazard ratio; VATS, video‐assisted thoracoscopic surgery; OT, open thoracotomy.

Figure 3 shows the pooled Kaplan–Meier curves for recurrence free survival. There is no significant difference between the VATS and OT groups (HR 1.07; 95% CI 0.88–1.29; *p* = 0.48; Figure 3 and Table 2).

**FIGURE 4:**
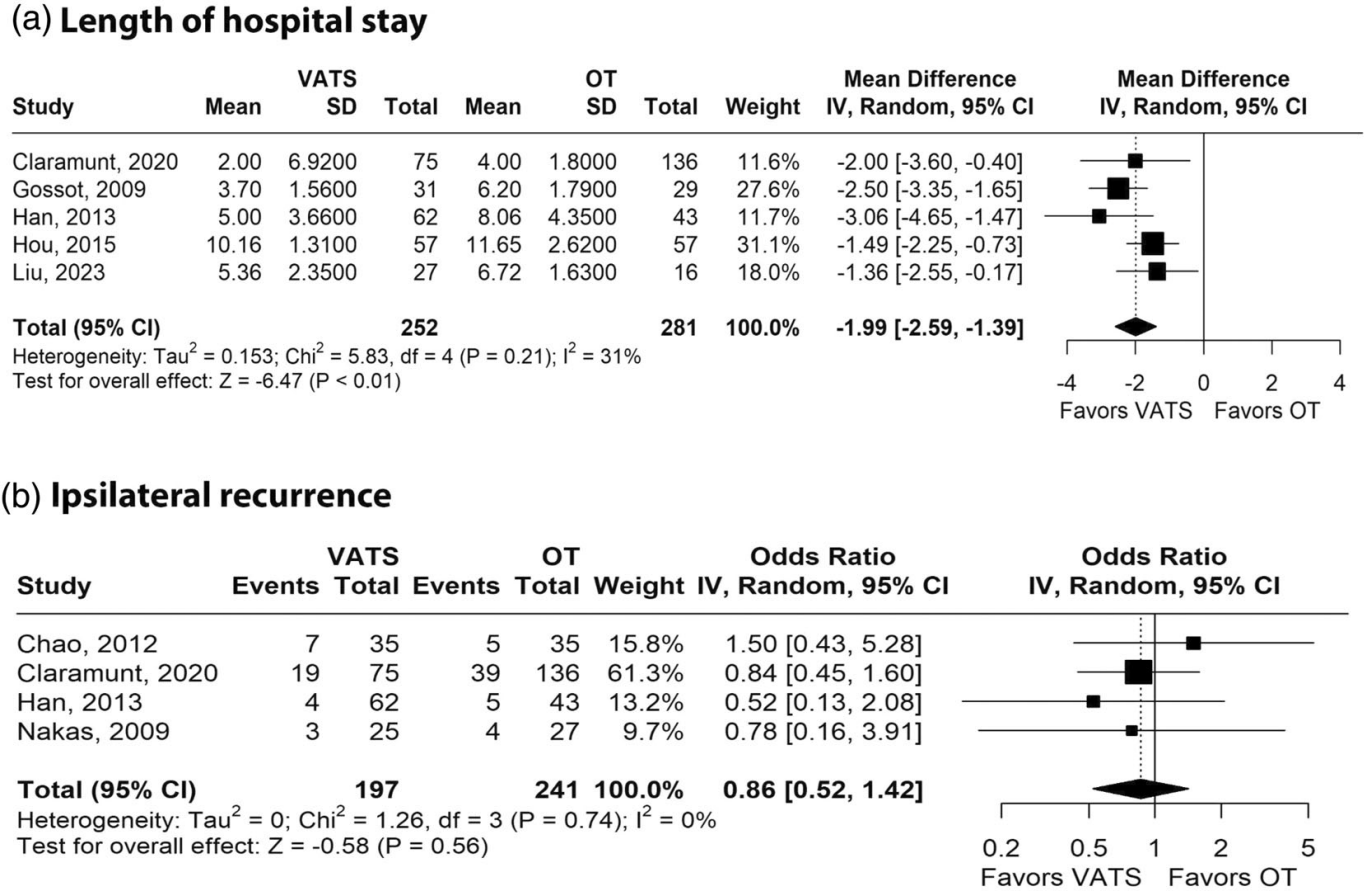
Forest plots of comparison between video‐assisted thoracoscopic surgery and open thoracotomy for pulmonary metastasectomy for patients underwent pulmonary metastasectomy. (a) Length of hospital stay. (b) Ipsilateral recurrence. CI, confidence interval; MD, mean difference; OR, odds ratio; OT, open thoracotomy; VATS, video‐assisted thoracoscopic surgery.

Figure 4a shows the forest plot for hospital LOS. Compared with the OT group, the VATS group presented significant shorter hospital LOS (MD −1.99 days; 95% CI −2.59 to −1.39; *I* ^2^ = 31%; *p* < 0.01; Figure 4a and Table 2).

Figure 4b shows the forest plot for ipsilateral recurrences. There is no significant difference between the VATS and OT groups (OR 0.86; 95% CI 0.52–1.42; *p* = 0.56; *I* ^2^ = 0%; Figure 4b and Table 2).

## DISCUSSION

In this systematic review and meta‐analysis of 11 studies including 2159 patients, we have comprehensively analyzed the outcomes of VATS versus OT in patients undergoing pulmonary metastasectomy. Our main findings were as follows: (1) patients undergoing VATS demonstrated enhanced overall survival compared to those in the OT group, (2) there was no significant difference in recurrence‐free survival between the VATS and OT cohorts, (3) patients who underwent VATS experienced a shorter hospital LOS relative to their OT counterparts, and (4) there was no significant difference in ipsilateral recurrence rates between the two groups.

Metastatic lung disease poses significant challenges.^1^, ^2^, ^3^, ^4^ Although surgical resection can improve survival,^1^, ^2^, ^4^, ^5^, ^6^, ^7^, ^8^, ^9^ the optimal approach (i.e., open thoracotomy or video‐assisted thoracoscopic surgery) remains debated due to concerns about margins and occult nodules related to VATS.^3^, ^5^, ^11^, ^13^, ^16^

Minimally invasive techniques offer benefits like reduced pain, faster recovery, and improved tolerance to postoperative oncological treatments.^2^, ^5^, ^12^, ^13^, ^14^ In our meta‐analysis, a clear advantage was observed in terms of overall survival for patients undergoing VATS. This aligns with the trend towards minimally invasive surgical approaches and highlights the potential benefits of VATS in improving overall survival outcomes for these patients.

Furthermore, because some studies included in our analysis were published during the earlier phases of VATS implementation, we conducted a subgroup analysis, categorizing the studies into two groups based on publication date: those prior to 2010 and those published thereafter. The results revealed a significant survival advantage for patients undergoing VATS compared to OT in newer studies. This suggests that advancements in VATS techniques, technology, and patient selection criteria may have contributed to improved outcomes in more recent studies. Conversely, there was no significant difference in overall survival for studies published before 2010. This indicates a temporal aspect to the effectiveness of VATS over time, highlighting the potential for improved surgical outcomes with ongoing advancements in minimally invasive surgical techniques.

Nevertheless, the efficacy of VATS heavily relies on precise preoperative imaging for nodule identification, as the impossibility of palpating the entire lung may lead to missed nodules and potentially increased recurrence rates.^4^, ^6^, ^7^, ^10^, ^11^, ^12^, ^13^, ^15^, ^16^ In contrast, Chen et al.^24^ demonstrated that advances in imaging technology have reduced the importance of palpation during OT, even for smaller lesions. Despite the higher number of nodules detected during OT, its impact on improving OS was uncertain. Our study indicates no significant difference in recurrence‐free survival or ipsilateral recurrence rates between the VATS and OT groups. This result suggests that while VATS may offer advantages in overall survival, its impact on recurrence rates may not be as pronounced as theorized.

In the early 2010s, two prospective studies were published. Cerfolio et al.^16^ reported that nearly 60% of nodules detected only by bimanual palpation were malignant. In contrast, Eckardt et al.^25^ found that approximately 65% of these nodules were benign. More recently, Liu et al.^5^ indicated that while the OT group had a higher number of resected malignant nodules, the overall survival rates were comparable between both surgical approaches. This discrepancy underscores the ongoing controversy regarding the benefits of resecting these lesions, as it may not lead to improved survival outcomes.

Additionally, patients undergoing VATS experienced a shorter hospital stay compared to those undergoing OT. This finding highlights the benefit of VATS in promoting faster recovery and reducing hospitalization periods.^2^, ^5^, ^12^, ^13^, ^14^ This finding is especially pertinent given the current healthcare environment, where optimizing patient outcomes while minimizing hospital stay lengths is crucial for maximizing bed availability for other patients awaiting surgical intervention. Lipińska et al.^26^ noted that while the costs associated with VATS are higher than those for OT, the overall hospitalization costs did not significantly differ between the two groups.

Finally, we identified two earlier meta‐analyses published on this topic in 2014^9^ and 2015.^4^ Despite our analysis encompassing a considerably larger sample size, our findings regarding overall survival and recurrence‐free survival outcomes are consistent with theirs. We did not uncover any prior meta‐analyses utilizing reconstructed time‐to‐event data for comparison.

### Study strength and limitations

This meta‐analysis is the first to reconstruct time‐to‐event data specifically to examine this important topic. However, there are several limitations to consider. The studies analyzed are predominantly retrospective, which introduces inherent selection bias and often includes incomplete documentation of treatments for primary malignancies. Additionally, the decision to use VATS or OT is often influenced by the surgeon's preference rather than the characteristics of the lesion or the patient. Thus, a treatment allocation‐bias inherent to the studies involved is a factor that is difficult to isolate. Ultimately, in the absence of RCTs, our findings nonetheless offer insights into the clinical applicability of VATS for patients with pulmonary metastases.

## CONCLUSION

For patients undergoing pulmonary metastasectomy, VATS strategy is associated with higher survival rates and reduced hospital LOS when compared with OT. Moreover, metastasis recurrence does not seem to be associated with long‐term mortality in this population.

## AUTHOR CONTRIBUTIONS

FP and TC designed the study. FP and PB performed the literature review. FP, LF, HK and TS selected the studies, qualified the studies according to the risk of bias, performed the data abstraction, built the tables, and organized the results. FP and TC performed the statistical analyses. PS and TD analyzed the data. FP, LF and TC wrote the manuscript. All the authors approved the final version of the manuscript.

## FUNDING INFORMATION

This work was supported by the Deutsche Forschungsgemeinschaft (DFG, German Research Foundation to Tulio Caldonazo) Clinician Scientist Program OrganAge funding number 413668513, by the Deutsche Herzstiftung (DHS, German Heart Foundation to Tulio Caldonazo) funding number S/03/23, and by the Interdisciplinary Center of Clinical Research of the Medical Faculty Jena.

## CONFLICT OF INTEREST STATEMENT

The authors declare no conflicts of interest.

## Supporting information


**Supplementary Table 1.** Complete search strategy.
**Supplementary Table 2.** Assessment of risk of bias using the Newcastle Ottawa Scale.
**Supplementary Figure 1.** Two‐stage meta‐analysis of the primary endpoint (overall survival).
**Supplementary Figure 2.** Leave‐one‐out analysis for the primary endpoint (overall survival).
**Supplementary Figure 3.** Funnel plot for the primary endpoint (overall survival).
**Supplementary Figure 4.** Subgroup analysis addressing studies published before and after 2010 for the primary endpoint (overall survival).

## Data Availability

The data underlying this article are available in the article and in its online supplementary material.
