# Supplementary material for: Video‐assisted thoracoscopic surgery versus open thoracotomy for resection of lung metastasis—A meta‐analysis of reconstructed time‐to‐event data
Source: Thorac Cancer. 2024 Oct 17;15(34):2401–7. doi: 10.1111/1759-7714.15473 (PMC11609047; doi:10.1111/1759-7714.15473)

**Supplementary Material**

**Supplementary Table 1.** Complete search strategy.

**Supplementary Table 2.** Assessment of risk of bias using the Newcastle Ottawa Scale.

**Supplementary Figure 1.** Two-Stage meta-analysis of the primary endpoint (overall survival).

**Supplementary Figure 2.** Leave-one-out analysis for the primary endpoint (overall survival).

**Supplementary Figure 3.** Funnel plot for the primary endpoint (overall survival).

**Supplementary Figure 4.** Subgroup analysis addressing studies published before and after 2010 for the primary endpoint (overall survival).

**Supplementary Table 1.** Complete search strategy.

| Search: ("pulmonary metastasis" OR "pulmonary metastases" OR "lung metastases" OR "lung metastasis" OR "secondary lung cancer" OR "metastasectomy" OR "metastatectomy" OR "metastectomy") AND (VATS OR RATS OR "video assisted thoracic surgery" OR "video-assisted thoracoscopic" OR "robotic assisted thoracic surgery" OR thoracoscopic OR thoracoscopy) AND (thoracotomy OR "open surgery" OR "thoracic surgery") |
| --- |
| ("pulmonary metastasis"[All Fields] OR "pulmonary metastases"[All Fields] OR "lung metastases"[All Fields] OR "lung metastasis"[All Fields] OR "secondary lung cancer"[All Fields] OR "metastasectomy"[All Fields] OR "metastatectomy"[All Fields] OR "metastectomy"[All Fields]) AND ("thoracic surgery, video assisted"[MeSH Terms] OR ("thoracic"[All Fields] AND "surgery"[All Fields] AND "video assisted"[All Fields]) OR "video-assisted thoracic surgery"[All Fields] OR "vats"[All Fields] OR ("rats"[MeSH Terms] OR "rats"[All Fields]) OR "video assisted thoracic surgery"[All Fields] OR "video-assisted thoracoscopic"[All Fields] OR "robotic assisted thoracic surgery"[All Fields] OR ("thoracoscopes"[MeSH Terms] OR "thoracoscopes"[All Fields] OR "thoracoscope"[All Fields] OR "thoracoscopic"[All Fields] OR "thoracoscopical"[All Fields] OR "thoracoscopically"[All Fields]) OR ("thoracoscopy"[MeSH Terms] OR "thoracoscopy"[All Fields] OR "thoracoscopies"[All Fields])) AND ("thoracotomy"[MeSH Terms] OR "thoracotomy"[All Fields] OR "thoracotomies"[All Fields] OR "open surgery"[All Fields] OR "thoracic surgery"[All Fields]) |
| **Translations** |
| **VATS:** "thoracic surgery, video-assisted"[MeSH Terms] OR ("thoracic"[All Fields] AND "surgery"[All Fields] AND "video-assisted"[All Fields]) OR "video-assisted thoracic surgery"[All Fields] OR "vats"[All Fields]  **RATS:** "rats"[MeSH Terms] OR "rats"[All Fields]  **thoracoscopic:** "thoracoscopes"[MeSH Terms] OR "thoracoscopes"[All Fields] OR "thoracoscope"[All Fields] OR "thoracoscopic"[All Fields] OR "thoracoscopical"[All Fields] OR "thoracoscopically"[All Fields]  **thoracoscopy:** "thoracoscopy"[MeSH Terms] OR "thoracoscopy"[All Fields] OR "thoracoscopies"[All Fields]  **thoracotomy:** "thoracotomy"[MeSH Terms] OR "thoracotomy"[All Fields] OR "thoracotomies"[All Fields] |

**Supplementary Table 2.** Assessment of risk of bias using the Newcastle Ottawa Scale.

| **Study** | **Selection** | **Comparability** | **Outcome/Exposure** |
| --- | --- | --- | --- |
| Carballo | ******** | ****** | ******* |
| Chao | ******** | ****** | ******* |
| Claramunt | ******** | ****** | ******* |
| Gossot | ******** | ****** | ******* |
| Han | ******** | ****** | ******* |
| Hou | ******** | ****** | ******* |
| Liu | ******** | ****** | ******* |
| Markowiak | ******** | ****** | ******* |
| Murakawa | ******** | ****** | ******* |
| Mutsaerts | ******** | ***** | ******* |
| Nakas | ******** | ****** | ******* |


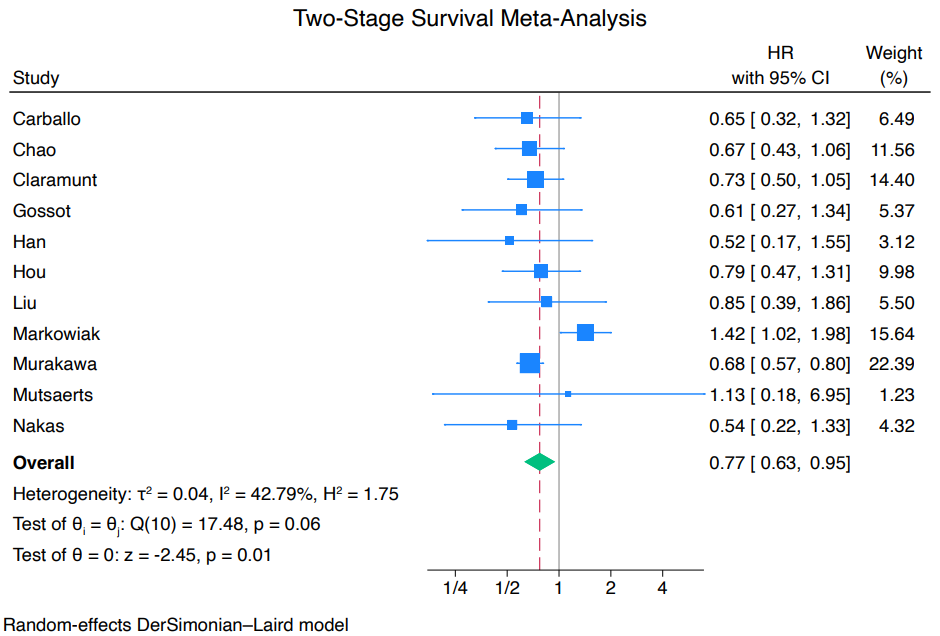
**Supplementary Figure 1.** Two-Stage meta-analysis of the primary endpoint (overall survival).

**Supplementary Figure 2.** Leave-one-out analysis for the primary endpoint (overall survival). **
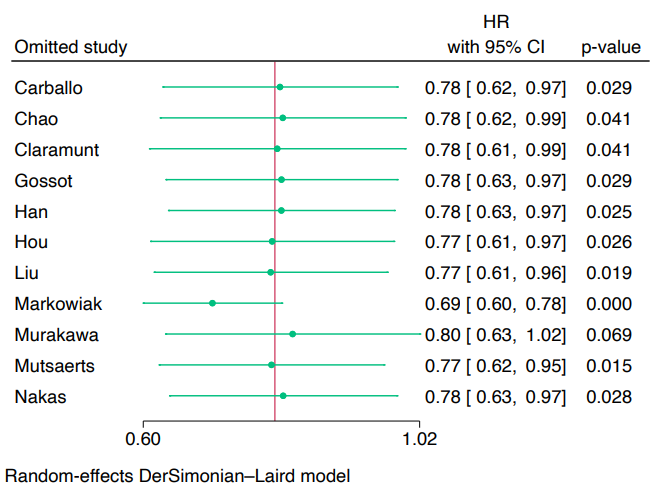
**


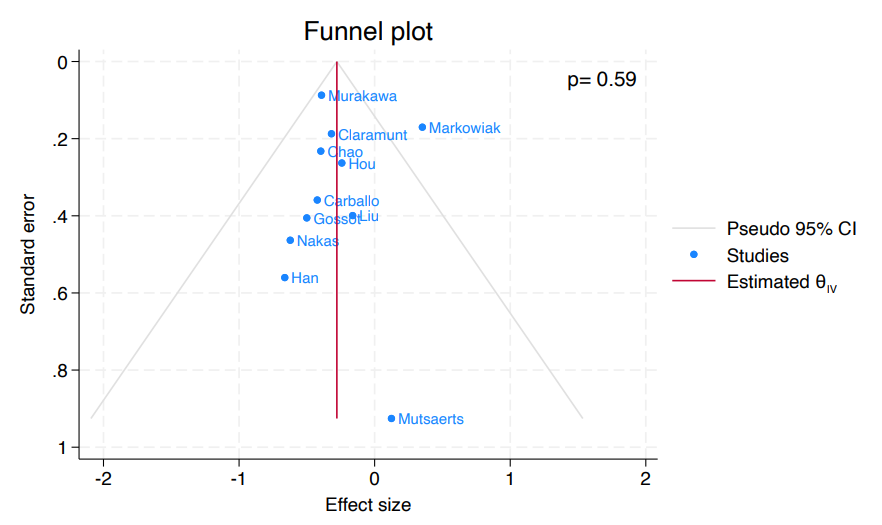
**Supplementary Figure 3.** Funnel plot for the primary endpoint (overall survival).

**Supplementary Figure 4.** Subgroup analysis addressing studies published before and after 2010 for the primary endpoint (overall survival).


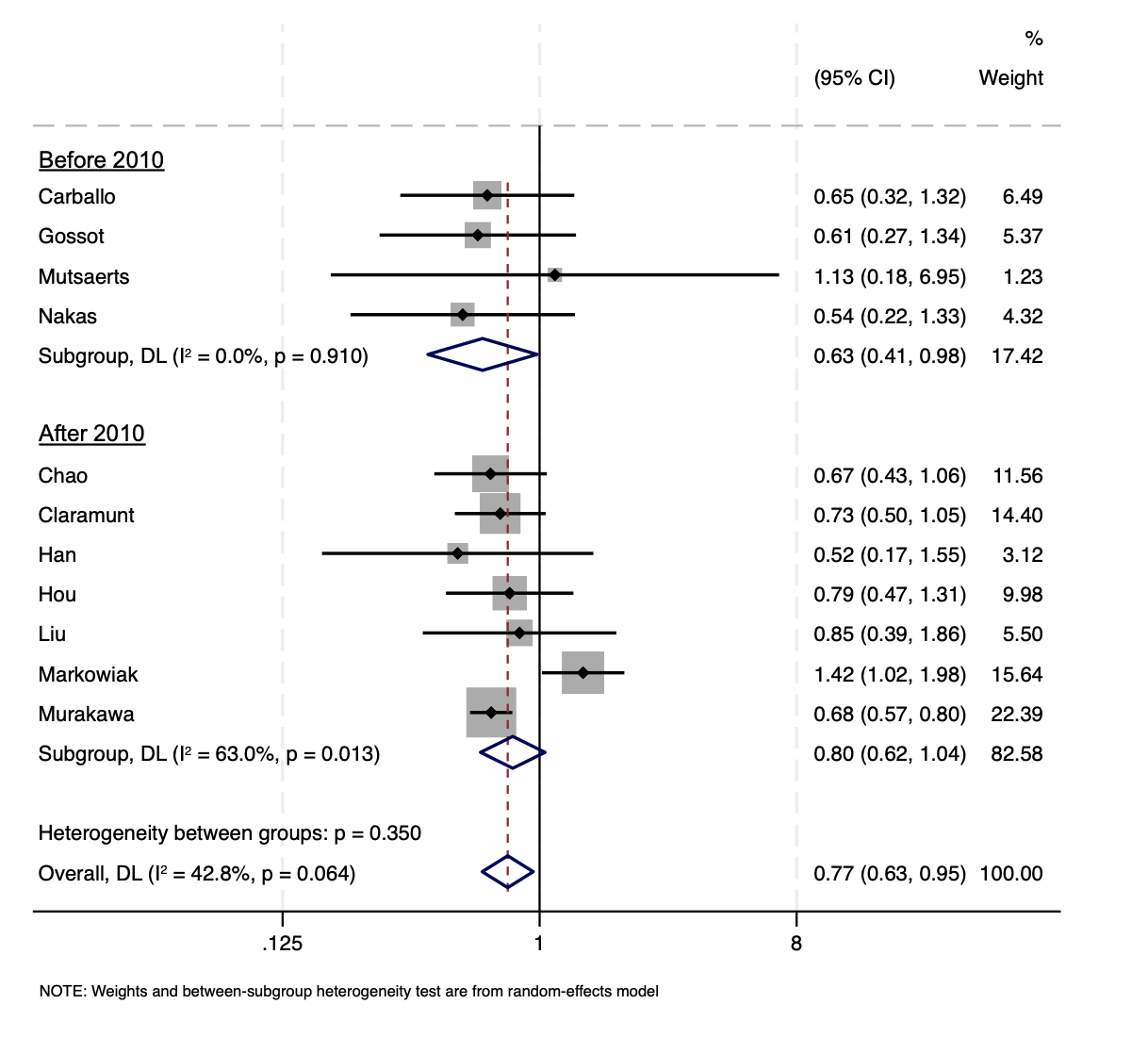

Supplement: Supplementary file 1 — Supplementary Table 1. Complete search strategy. Supplementary Table 2. Assessment of risk of bias using the Newcastle Ottawa Scale. Supplementary Figure 1. Two‐stage meta‐analysis of the primary endpoint (overall survival). Supplementary Figure 2. Leave‐one‐out analysis for the primary endpoint (overall survival). Supplementary Figure 3. Funnel plot for the primary endpoint (overall survival). Supplementary Figure 4. Subgroup analysis addressing studies published before and after 2010 for the primary endpoint (overall survival). [file TCA-15-2401-s001.docx]
